# Supplementary material for: What does it take to learn the rules of RNA base pairing? A lot less than you may think
Source: bioRxiv. 2025 Aug 2:2025.07.31.668042. Preprint. [Version 1] doi: 10.1101/2025.07.31.668042 (PMC12324431; doi:10.1101/2025.07.31.668042)
Supplement: Supplement 1 [file media-1.gz › supplemental_material/src/rscape_v2.6.0.tar.gz!/rscape_v2.6.0/lib/hmmer/documentation/userguide/inclusions/domain-serialize.pdf]

<table>
<tr><td>Size (4 bytes)</td><td>Ienv (8 bytes)</td><td colspan="2">Jenv (8 bytes)</td></tr>
<tr><td colspan="2">Iali (8 bytes)</td><td colspan="2">Jali (8 bytes)</td></tr>
<tr><td colspan="2">Iorf (8 bytes)</td><td colspan="2">Jorf (8 bytes)</td></tr>
<tr><td>Envsc (4 bytes)</td><td>Domcorrection (4 bytes)</td><td>Dombias (4 bytes)</td><td>Oasc (4 bytes)</td></tr>
<tr><td>Bitscore (4 bytes)</td><td colspan="2">lnP (8 bytes)</td><td>Is_reported (4 bytes)</td></tr>
<tr><td>Is_included (4 bytes)</td><td colspan="3">Scores_per_pos length (4 bytes)</td></tr>
<tr><td colspan="4">Scores_per_pos array (Variable # of elements, 4 bytes/element)</td></tr>
<tr><td colspan="4">ad (P7_Alidisplay, Variable Length)</td></tr>
</table>
